# NLRP3 Inflammasome/Pyroptosis: A Key Driving Force in Diabetic Cardiomyopathy

**DOI:** 10.3390/ijms231810632

**Published:** 2022-09-13

**Authors:** Lixia Zhang, Chenchen Ai, Ming Bai, Jinglei Niu, Zheng Zhang

**Affiliations:** 1 The First School of Clinical Medicine, Lanzhou University, Lanzhou 730000, China; 2 Department of Heart Center, The First Hospital of Lanzhou University, Lanzhou 730000, China; 3 Cardiovascular Center, Gansu Provincial Maternity and Child-Care Hospital, Lanzhou 730050, China

**Keywords:** NLRP3 inflammasome, pyroptosis, diabetic cardiomyopathy, inhibitor

## Abstract

Diabetic cardiomyopathy (DCM), a serious diabetic complication, is a kind of low-grade inflammatory cardiovascular disorder. Due to the high risk of morbidity and mortality, DCM has demanded the attention of medical researchers worldwide. The pathophysiological nature of DCM is intricate, and the genesis and development of which are a consequence of the coaction of many factors. However, the exact pathogenesis mechanism of DCM remains unclear. Pyroptosis is a newly identified programmed cell death (PCD) that is directly related to gasdermin D(GSDMD). It is characterized by pore formation on the cell plasma membrane, the release of inflammatory mediators, and cell lysis. The initiation of pyroptosis is closely correlated with NOD-like receptor 3 (NLRP3) activation, which activates caspase-1 and promotes the cleaving of GSDMD. In addition to adjusting the host’s immune defense, NLRP3 inflammasome/pyroptosis plays a critical role in controlling the systemic inflammatory response. Recent evidence has indicated that NLRP3 inflammasome/pyroptosis has a strong link with DCM. Targeting the activation of NLRP3 inflammasome or pyroptosis may be a hopeful therapeutic strategy for DCM. The focus of this review is to summarize the relevant mechanisms of pyroptosis and the relative contributions in DCM, highlighting the potential therapeutic targets in this field.

## 1. Introduction

Diabetic cardiomyopathy is characterized by the cardiac insufficiency that exists in diabetic individuals, and which is distinguished from other common heart diseases. DCM has been regarded as a severe complication of diabetes, which is closely related to poor prognoses. Previous data have shown that the incidence of heart failure in diabetic individuals increases independent of accompanied hypertension and myocardial infraction [1]. The definition of DCM was originally proposed to describe ventricular dysfunction in diabetics without comorbid coronary heart disease and hypertensive disease [2]. However, currently, it is used more widely to describe increased susceptibility to myocardial injury and dysfunction in diabetic individuals. The clinical features of DCM are generally considered to include four stages, and each stage represents different points of disease progression [3]. In the first stage, the patients are often asymptomatic, except cardiac diastolic dysfunction. In stages 2 and 3, the affected individuals are characterized by significant systolic and diastolic dysfunction. In the final stage, patients present with typical heart-failure symptoms, as well as structural changes featured with cardiac dilatation and fibrosis, which can often be confirmed by late gadolinium enhancement (LGE) imaging in cardiac magnetic resonance (CMR) [4,5]. Additionally, in this stage, coronary artery disease is easily combined. Given the diversity of its clinical manifestations, the early diagnosis and management of DCM is challenging. The molecular mechanism of DCM is complicated, which is composed of multiple factors combined [6]. Lately, the underlying mechanism of programmed cell death caused by the inflammation in DCM has been drawing growing attention.

The concept of cell death was first put forward in the 1960s. It is indispensable for sustaining the host’s homeostasis and regulating the development of diseases. Programmed cell death (PCD) drives the clearance of nonfunctional, infected, and even neoplastic cells. In the past few decades, apoptosis was regarded as the only pathway for PCD; however, recently, several new-style pathways have been identified, such as pyroptosis, necroptosis, and ferroptosis, which have been demonstrated to play a critical part in both physiological and pathological conditions. Pyroptotic cell death was first observed in macrophages in 1992, after studies showed its rapid lysis after being infected with *Shigella flexneri* [7]. Until 2001, the term “pyroptosis” was proposed for the first time and recognized as a new type of PCD depending on caspase-1 [8]. The trigger of pyroptosis is strongly linked to NLRP3 inflammasome, which activates downstream molecules to prompt the rupture of membranes and the production of mature pro-inflammatory cytokines. An accumulating body of studies indicates that pyroptosis participates in the pathogenesis of diversified cardiovascular diseases (CVDs), including atherosclerosis, ischemia–reperfusion injury, and diabetic cardiomyopathy [9,10,11,12,13,14]. Numerous pre-clinical research has illuminated that pyroptosis is highly linked with the genesis and progression of DCM, whether in vivo or in vitro. Consequently, designing therapeutic strategies that target pyroptosis may give new avenues of treatment of DCM.

Herein, we summarize the present state of progress and development trends in pyroptosis and related inflammasome research and especially highlight its role in diabetic cardiomyopathy. Furthermore, we highlight the emerging pharmacological approaches of DCM that target the pyroptosis and related inflammasomes, which might be helpful options in clinical therapeutics.

## 2. NLRP3 Inflammasome

The primary function of innate immunity is to defend against pathogen aggression and maintain homeostasis. Inflammasomes are macromolecular complexes, which belong to the pattern-recognition receptor (PRR) family, constituting a vital part of innate immunity. Inflammasomes are assembled by inflammatory caspases and various inflammasome sensors including NLRs (NOD-like receptors) and pyrin, as well as AIM2 (absent in melanoma2-like receptors) [15,16,17]. The NLRs family members all have a central nucleotide-binding and oligomerization (NACHT) domain and a C-terminal leucine-rich repeat (LRR) domain, as well as a respective N-terminal domain [15]. The NLR family has many members, containing NLRP1, NLRP3, NLRP6, and NLRC4 [18,19]. The best characterized inflammasome is the NLRP3 inflammasome.

**Figure 1:**
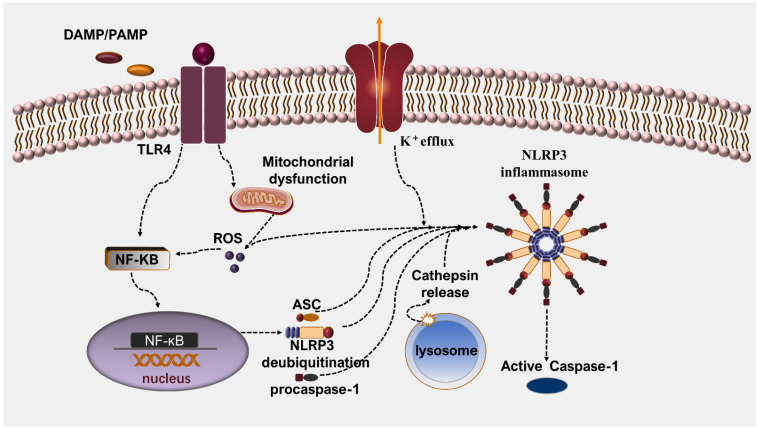
The mechanism of NLRP3 inflammasome activation. The process of activation contains two phases: priming and activating. In the priming phase, DAMP or PAMP induces the activation of NF-κB signal and triggers an increase in NLRP3 expression. Additionally, NLRP3 deubiquitnation, the adaptor molecule ASC, and procaspase-1 together accomplish the assembly of inflammasome. In the activating phase, several distinct stimuli signals are required to activate NLRP3 inflammasomes, such as mitochondrial ROS, K^+^ efflux, and cathepsins released by lysosome.

The NLRP3 inflammasome consists of NLRP3, adaptor ASC (apoptosis-associated speck-like protein containing a caspase activation and recruitment domain), and pro-caspase-1. ASC contains a two-structure domain, pyrin and CARD (caspase activation and recruitment domain). Through CARD-CARD interaction, ASC can couple upstream PRRs to caspase-1 [20]. Then, pro-caspase-1 can generate caspase-1*p*10/*p*20 subunits through a series of self-cleavage [21]. For one thing, caspase-1 can cleave proIL- 1*β* and proIL-18 into their activated forms [22,23]. Meanwhile, caspase-1 promotes GSDMD to translate into GSDMD-CT (C-terminal domain) and GSDMD-NT (GSDMD N-terminal domain), causing membrane lysis to trigger pyroptosis [24]. Two phases are contained in the process of NLRP3 inflammasome activation. The mechanisms of action related to the two phases of the NLRP3 inflammasome are shown in Figure 1. In the priming phase, through the activation of nuclear factors-κB (NF-κB), agonists activate the TLR4 receptors and augment the gene production of NLRP3 and pro-inflammatory mediators. On the basis of recognizing dangerous signals, once the inflammasome assembly is completed, pro-caspase-1 is encouraged to cleave into its active form and initiates consequent reactions [20,25,26,27]. In the activating phase, several distinct stimulus signals are required. Numerous stimuli that can activate NLRP3 inflammasomes have been identified, including pathogens, endogenous stimulus, and environmental particular matter [28,29,30,31]. Up to the present moment, three theories of NLRP3 inflammasome activation have been accepted. The first theory is that extracellular ATP irritates the cytomembrane-based P2X7 (purinergic receptor P2X, ligand-gated ion channel 7) and causes the potassium efflux. The low level of K^+^ can directly result in the activation of the NLRP3 inflammasome [32,33]. The second theory is the lysosome rupture model. Under this circumstance, crystalline materials such as cholesterol crystal, silica crystals, and aluminum salts can induce lysosomal damage via crystal absorption and the release of cathepsin B. Subsequently, this results in NLRP3 inflammasome activation [34,35,36]. In the third theory, mitochondrial injury and an increase in reactive oxygen species (ROS) can activate the NLRP3 inflammasome [37,38,39]. However, it is worth noting that, after being infected by RNA viruses, the production of IL-1*β* can be reduced by a reduction in mitochondrial membrane potential (ΔΨ(m)). This suggests that the NLRP3 inflammasome can be excited in a ΔΨ(m)-dependent manner [40]. Therefore, the correlation of mitochondrial dysfunction and NLRP3 inflammasome needs further in-depth exploration.

Moreover, NEK7 is a crucial requirement during NLRP3 inflammasome activation. NIMA-related kinase 7 (NEK7) is a mitotic Ser/Thr kinase belonging to the NERKs family. In addition to a N-terminal end, the structure of NERK7 is characterized as a central kinase domain [41]. As described previously, the K^+^ efflux is a committed step for activating the NLRP3 inflammasome [33,42]. NEK7 can directly act with downstream potassium efflux and can regulate NLRP3 activation, which further elucidates the mode of action between NLRP3 inflammasome activation and K^+^ efflux [43].

## 3. Pyroptosis

Pyroptosis is distinguished from any other PCD, regardless of the morphology and mechanism. The major difference between pyroptosis and apoptosis is the involvement of caspases. The caspases involved in apoptosis include the initiation caspases and the executioner caspases. The former contains caspase-2,8,9,10, and caspase-3,6,7 belong to the latter. None of them exist in the process of pyroptosis [44,45]. In addition, the phenomenon of plasma membrane pore-formation caused by GSDMD-NT is absent in apoptosis [46].

**Figure 2:**
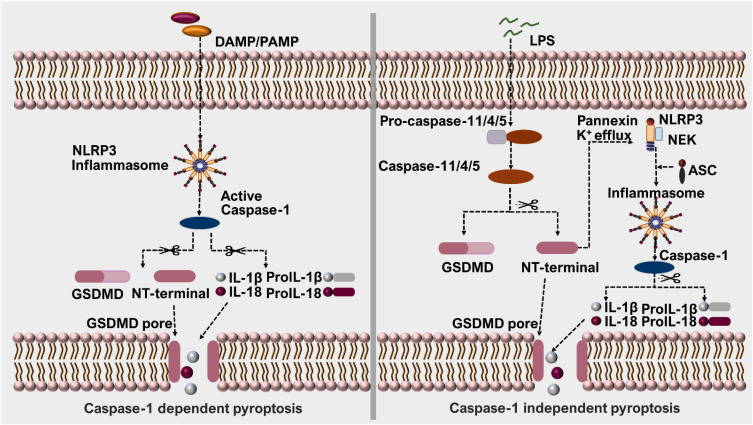
The mechanism of caspase-1 dependent and independent pathway. In the caspase-1-dependent pathway, DAMP and PAMP act by cleaving GSDMD, whilst GSDMD-NT directly mediates the pore formation of cell membranes. In the caspase-1-independent pathway, caspase-11/4/5 are activated by LPS released from Grams-negative bacteria to mediate pyroptosis. GSDMD-NT generated from this process can also boost NLRP3 inflammasome activation and further induction of pyroptosis.

It has been confirmed that there are two signal pathways for pyroptosis, caspase-1 dependent and independent pathway (Figure 2). In humans, caspase-4/5 are required in the pyroptotic non-canonical pathway, whereas caspase-11 is essential for mice [47,48]. It is vital to activate the respective caspases for different pathways. The canonical pathway of pyroptosis relies on the activation of caspase-1 (Figure 2) [49].

### 3.1. Caspase-1-Dependent Pathway

As noted before, once NLRP3 inflammasome is activated, the NLRs oligomerize and subsequently recruit adopt-proteins ASC. After that, a huge speck-like structure is made up of ASC and recruits pro-caspase-1 via CARD-CARD interplay. Pro-caspase-1 can self-cleave into its active form and then regulate the process of proinflammatory cytokines [50,51]. Meanwhile, active caspase-1 transforms GSDMD into GSDMD-NT, inducing the formation of cell membrane pores [52,53]. GSDMD is a downstream molecule of inflammatory caspases. The ultrastructure of GSDMD was confirmed in 2019 in both humans and mice [54]. As a final executor of downstream inflammasome activation, the cleavage of GSDMD induced by caspases occurs at the N-terminal and C-terminal junction [55]. The integrity of the cell membranes, containing cardiolipin and phosphatidylinositol phosphates, will be disrupted and followed by pyroptosis when combined with GSDMD-NT [52,53,56]. By means of single-cell analysis technology, Nathalia M.de Vasconcelos and his colleagues revealed that subcellular events induced by GSDMD occur prior to the disruption of plasma membranes during the process of pyroptosis [57].

### 3.2. Caspase-1-Independent Pathway

In the non-canonical pathway, caspase-11 or caspase-4/5 are required for initiating pyroptosis, respectively (Figure 2). In this pathway, pyroptosis and the production of inflammatory cytokines are induced by several Gram-negative bacteria [58,59]. Lipopolysaccharide (LPS) is a endotoxin, belonging to prototypic pathogen-associated molecular patterns (PAMP), which mainly exist in the outer walls of cell membranes in major Gram-negative bacteria [60]. The host’s guanylate-binding protein (GBP) recruitment to Gram-negative bacterial out membranes is driven by LPS, which is a crucial step in the initiation of caspase-11 activation [61]. Kayagaki et al. revealed that the deletion of caspase-11 saved mice from the damage caused by a lethal dose of LPS, which is absent in caspase-1. It is highlighted that caspase-11 makes a significant impact in severe inflammatory response [62]. Being a specific substrate for the above inflammatory caspases, GSDMD is usually cleaved at D276/G277 sites, generating GSDMD-NT and GSDMD- CT, and resulting in pyroptosis [63]. Meanwhile, pannexin-1 and GSDMD are considered to mediate K^+^ efflux for activating NLRP3 inflammasome in this pyroptotic pathway [64,65].

## 4. NLRP3 Inflammasome/Pyroptosis in Diabetic Cardiomyopathy

Over the last several decades, the morbidity of diabetes has been surging continuously, and this disorder has become the most prevalent disease worldwide. Cardiovascular disorder is the primary reason of death in affected individuals. In advanced type 2 diabetic mellitus, cardiac dysfunction is the pervasive clinical manifestation. This phenomenon was termed as diabetic cardiomyopathy (DCM), described in a small cohort of four patients for the first time in 1972, which is not associated with other common cardiovascular diseases [2]. Structural impairment and cardiac dysfunction are both hallmarks of DCM; however, affected individuals do not manifest with many symptoms in the early stage [66]. Diastolic dysfunction is the major feature of DCM in the early stages, which has been well-established by clinicians and scholars [6,67,68]. In the later stage, cardiac hypertrophy and fibrosis are the prototypical properties of DCM [68,69].

### 4.1. Mechanism of NLRP3 Inflammasome/Pyroptosis in DCM

**Figure 3:**
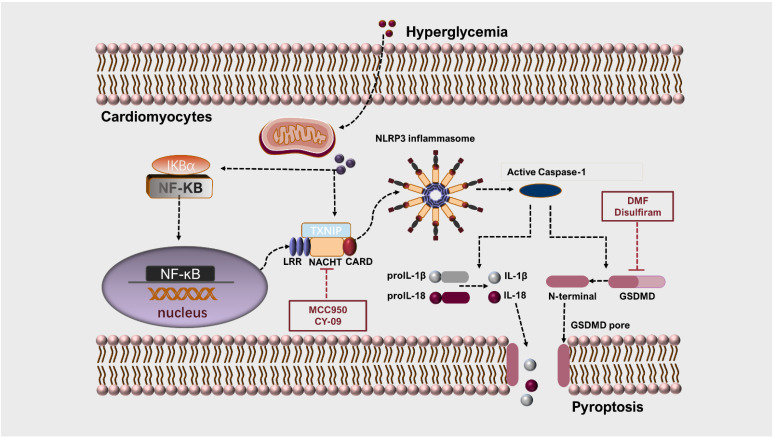
Mechanisms of NLRP3 inflammasome and pyroptosis in DCM. Taking cardiomyocytes as an example, hyperglycemia increases the generation of intracellular ROS, induced by mitochondrial dysfunction, which causes the separation of TXNIP from TRX and binds to NLRP3. This process results in NLRP3 inflammasome activation and pro-caspase-1 autocleavage. Additionally, ROS activates NF-κB, promoting the construction of molecular platform for NLRP3 inflammasome and self-cleavage of caspase-1, then facilitates the procession of IL-1*β* and IL-18. Activated caspase-1 also promotes GSDMD cleavage into its active form, subsequently inducing membrane pore formation and leading to cellular swelling and pyroptosis. CY-09 and MCC950 are the special inhibitors of NLRP3, which can exert an inhibitory effect by combining directly into ATP-binding motif of NACHT domain and restraining the ATPase activity of NLRP3. DMF can respond to GSDMD at a pivotal cysteine residue to promote GSDMD succination, then affects the interaction between GSDMD and caspases and consequent reactions. Disulfiram, a potent inhibitor of GSDMD, can abolish the formation of plasma membranes pore induced by GSDMD.

The pathophysiological mechanisms of DCM are very intricate, which is the outcome of the joint action of multiple factors and mechanisms such as inflammation, oxidative stress, mitochondria dysfunction, the information of AGEs (advanced glycation end products), and cell death [70,71,72,73,74]. At present, the cell death associated with DCM, especially pyroptosis, has aroused widespread concern among scholars [75,76]. Observed under an electron microscope, dying myocardium cells, both in diabetic rats and mice, exerted features of mitochondrial and fibrous swelling, which similarly occur in pyroptosis [77]. As a main contributor, the NLRP3 inflammasome drove the progression of DCM in rat models as well as the secretion of proinflammatory cytokines [78]. NF-κB, a principle mediator of inflammation, would be activated when the myocardium is exposed to high glucose or fatty acid, which can induce NLRP3 inflammasome activation as well as the secretion of pro-inflammatory mediators [75]. Surprisingly, inhibiting NF-κB can exacerbate the inflammation mediated by NLRP3 [79]. Thus, in-depth research targeting the interworking of NF-κB and NLRP3 is still indispensable. Thioredoxin-interacting protein (TXNIP), a specific inhibitor of thioredoxin (TRX), exerts a critical effect on regulating the intracellular redox reaction. In the hyperglycemic context, TXNIP expression is increased. Increased ROS generation due to high glucose can cause the TXNIP to separate from the TRX and combine with the NLRP3 [80]. The expressions of TXNIP and NLRP3 are both increased in type 2 diabetic patients’ circulating immune cells [81]. The mechanism of NLRP3 inflammasome activation induced by high glucose is detailed in Figure 3.

### 4.2. NLRP3 Inflammasome/Pyroptosis Activation in Different Cells in Diabetic Heart

**Figure 4:**
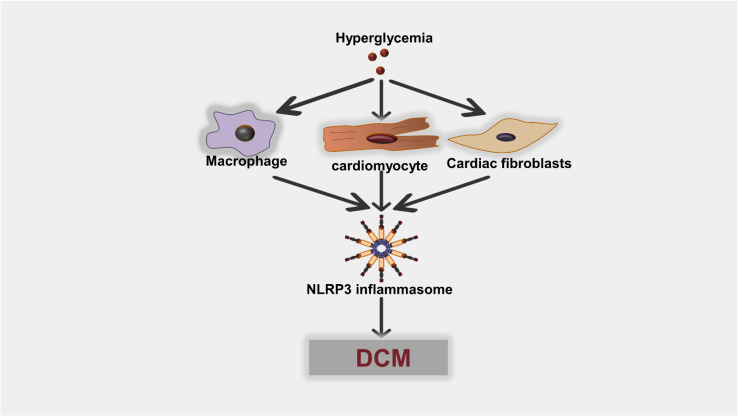
Hyperglycemia can cause NLRP3 inflammasome activation in different types of cells in the heart. Consequently, NLRP3 inflammasome activation and subsequent pyroptosis result in the occurrence of diabetic cardiomyopathy (DCM).

Pyroptosis can occur in multiple types of cells, including cardiac fibroblasts, cardiomyocyte, and macrophages, in the heart once under hyperglycemic stress or hyperlipidemia, which further contributes to the genesis and development of DCM (Figure 4). Cardiac fibroblasts (CFs) can be activated to convert into myofibroblasts when encountering various stimuli, which occurs in diabetic cardiomyopathy [82,83]. Published data show that pyroptosis partook in the process of cardiac fibrosis. Inflammasome in CFs can be activated when ROS production increases or potassium efflux occurs, then facilitating the production of IL-1β and IL-18 [84]. In primary myocardial fibroblasts extracted from neonatal SD rats, NLRP3 and its downstream proteins can be activated when treated with a high glucose concentration [85]. Similarly, pyroptosis also occurs in cardiomyocytes. A recent publication explained that NLRP3 inflammasome activation increased after H9C2 cardiomyocytes were stimulated with high glucose (HG) and hypoxia/reoxygenation (H/R); moreover, pyroptotic cell injury can be alleviated by ROS scavengers, inflammasomes, and caspase-1 inhibitors [86]. Under this condition, pyroptosis can be aggravated by LPS [87].

It is worth mentioning that macrophage pyroptosis participates in the pathogenesis of DCM. Macrophages are central regulators in the immune system; they can recognize, phagocytose and remove apoptotic cells, thereby initiating the immune response. Previously, clinical studies have shown that the levels of monocytes and proinflammatory cytokines increased in the peripheral blood of prediabetic cardiomyopathy patients [88,89]. Similar findings have been observed in preclinical studies. The infiltration of monocytes and macrophages existed in the myocardium in a model of diabetes, regardless of being type 1 or type 2 [90,91]. According to one recent study, M1 macrophage infiltration and NLRP3 inflammasomes both increased in cardiac ventricular muscles in mice models of type 2 diabetes after stroke. Even more interesting is that the NLRP3 inhibitor CY-09 can improve the cardiac function of diabetic mice [92]. This indicates that M1-macrophage polarization and NLRP3 inflammasome activation may have a significant impact on the brain-heart interaction in diabetes stroke. Bruton’s tyrosine kinase (BTK), a key receptor in B cell signal transduction, usually play a crucial part in the genesis of hematological tumors [93]. BTK also possesses the function of regulating inflammation and highly expresses in monocytes/macrophages. More recently, a new study presented that BKT took part in the regulation of the NLRP3 inflammasome and the production of proinflammatory cytokines, regardless of high-fat-diet chronic-inflammation mice models or human macrophages processed by LPS and ATP [94]. This means that NLRP3 inflammasome activation and the generation of correlative inflammatory factors in macrophages are closely associated with metabolic condition inflammation, especially in diabetes. To sum up, the NLRP3 inflammasome and its induced pyroptosis have a high correlation with the genesis and development of DCM. However, these studies related to DCM are still in their infancy, and there are many questions that need to be explored in depth.

### 4.3. The Role of NLRP3 Inflammasome/Pyroptosis on Cardiac Vasculature in DM/DCM

Macro- and micro-vessel injuries induced by diabetes are the primary cause of morbidity and mortality in diabetic individuals [95]. Diabetes mellitus is established as an independent risk factor for coronary artery diseases. Some studies have found that NLRP3 inflammasome/pyroptosis participated in the occurrence and development of coronary artery diseases [96,97]. NF-κB is highly expressed in many inflammatory diseases, including atherosclerosis (AS), which can induce endothelial dysfunction by promoting the production of inflammatory mediators [36]. Cyclooxygenase 2(COX-2), a downstream molecule of NF-κB, is also involved in the genesis and progression of atherosclerosis [98]. Intriguingly, COX-2 can positively regulate the activation of NLRP3 inflammasome and maturation of proinflammatory cytokines [99]. Cholesterol crystals play an important role in atherosclerotic plaque formation, which can directly activate the NLRP3 inflammasome by inducing the lysosome rupture and subsequent release of cathepsin B [100]. Other scholars have found that oxidized low-density lipoprotein (oxLDL) can induce NLRP3 inflammasome activation and inflammatory cytokines released in vascular endothelial cells via miR-125a-5p/TET2 pathway, promoting the development of AS [9]. However, there are few studies on the mechanism of NLRP3 inflammasome/pyroptosis in AS combined with diabetic cardiomyopathy.

Diabetes-specific factors can accelerate atherosclerotic plaque rupture and thrombosis, leading to myocardial infarction [101]. It is now well recognized that NLRP3 inflammasome/pyroptosis is implicated in the pathological process of acute myocardial infarction (AMI). Kawaguchi et al. demonstrated that ASC and caspase-1 were markedly expressed in infiltrated inflammatory cells of the ischemic myocardium [84]. Furthermore, they also reported that inflammasome activation induced by H/R existed in cardiac fibroblasts but not cardiomyocytes. Additional data show that the infarct size and inflammatory response were reduced in myocardial infarction models by using pharmacological and genetic inhibitions of NLRP3 [102,103]. Glucose variability (GV) means a measure of fluctuations in glucose or other homeostasis-related parameters of glucose over a given time interval, which has drawn much attention from scholars [104]. More recently, an observational study confirmed that high GV is an independent predictor of recurrent acute myocardial infarction (RAMI) [105]. The authors further found that high GV was closely linked to increased NLRP3 expression and the decreased expression of autophagy marker LC3B as well as stress-related proteins and GTPase-activating protein (SH3 domain)-binding-protein 1 (G3BP1). This indicates that GV can affect atherosclerotic development and rupture by mediating autophagy and the G3BP1/NLRP3 inflammasome pathway. However, to date, studies on AMI under the context of diabetic cardiomyopathy are scarce. Therefore, more attention is required in this area.

## 5. Potential Therapeutic Strategies for Targeting NLRP3 Inflammasome/Pyroptosis in DCM

Diabetic cardiomyopathy has been identified for several decades, but targeted therapeutic measures are still limited, relying mainly on hypoglycemic drugs. Although these drugs offer some cardiovascular protection, morbidity and mortality from DCM continue to climb. Therefore, there is an urgent need to develop specialized drugs for DCM to change this situation. As research continues, some substances were found to have good potential for the treatment of DCM by targeting the NLRP3 inflammasome or pyroptosis. This certainly brings new hope to clinicians and patients. These include classical antidiabetic drugs, phytochemicals, inhibitory compounds, and non-coding RNAs (ncRNAs) as well as some special proteins.

### 5.1. Hypoglycemic Agents

Accumulating evidence suggests that a variety of hypoglycemic agents possess the favorable properties of inhibiting the NLRP3 inflammasome activation or pyroptosis, thereby realizing the improvement of diabetic cardiomyopathy. These kinds of hypoglycemic drugs include metformin, peroxisome proliferator-activated receptors (PPARs) agonist, sodium-glucose cotransporter 2(SGLT2) inhibitors, and dipeptidyl peptidase-4 (DPP4) inhibitor.

Metformin is a kind of biguanide, a drug derived from herbs, applied as a first-line therapy for type 2 diabetes [106]. Metformin can downregulate the expressions of NLRP3, caspase-1, and proinflammatory cytokines in diabetic mice models by the AMPK (AMP-activated protein kinase) signal, thus achieving the improvement of DCM [107]. In STZ (streptozotocin)-induced hyperglycemia and hyperlipidemia in apoE ^−/−^ mice, metformin can suppress the development of diabetes-accelerated atherosclerosis and NLRP3 inflammasome activation, and this inhibitory effect can be abolished by AMPK inhibitor compound C [108]. Furthermore, metformin can protect against ischemic myocardial injury by mitigating the autophagy-ROS-NLRP3-signalling inflammation in macrophages [109]. These represent the direct proof that metformin can be used for the therapy of DCM through the suppression of NLRP3 inflammasome and pyroptosis.

SGLT2 inhibitor is a new type of antidiabetic agent, that reduces serum glucose by increasing urinary glucose excretion [110]. Dapagliflozin, a representative drug of sodium-glucose cotransporter-2 inhibitor (SGLT2i), has been widely applied to T2D patients. Recently, many clinical trials have verified that dapagliflozin can lower cardiovascular mortality and the heart failure hospitalization rate [111,112]. It can also reduce left ventricular masses and ameliorate the insulin resistance in the left ventricular hypertrophy that is accompanied with T2D [113]. Contemporaneous studies in animal models demonstrated that dapagliflozin, combined with ticagrelor, a P2Y12 antagonist, had a synergistic effect on attenuating the activation of NLRP3 inflammasome through the AMPK/mTOR axis for improving the progression of DCM [114]. Another investigation revealed that dapagliflozin alleviated cardiac fibrosis and left ventricular systolic function in ob/ob mice, as well as pyroptosis in high-glucose-treated CFs by inhibiting NLRP3 inflammasome activation. In addition, dapagliflozin and saxagliptin, a dipeptidyl peptidase-4 (DPP4) inhibitor, both have an additive effect on the above-mentioned models [115]. As shown above, we can draw a conclusion that dapagliflozin could diminish the progression of DCM by suppressing the NLRP3 inflammasome activation and subsequent pyroptosis. This protective action could be amplified by other antidiabetic agents, for example, DPP4 inhibitors. Empagliflozin, another kind of SGLT2 inhibitor, has been confirmed to have an association with the reduction in cardiovascular-related mortality and hospitalizations in type 2 diabetic patients [116]. Moreover, empagliflozin can also exert a cardioprotective effect in the absence of diabetes. In heart failure (HF) rodent models, induced by transverse aortic constriction surgery, empagliflozin can suppress NLRP3 inflammasome and the expression of associated inflammatory markers [117]. This beneficial effect of empagliflozin can be weakened by Ca^2+^ ignophore. Moreover, empagliflozin can reduce the cardiac fibrosis and inflammation in doxorubicin-induced cardiotoxicity mouse models through the NLRP3 and MyD88 signal pathway [118]. Of note, there are different points of view on this subject. Some researchers found that neither empagliflozin nor liraglutide exerted inhibitory effects on the activation of NLRP3 and downstream proinflammatory cytokines, despite the level of HbAc1 being attenuated [119]. Therefore, the concrete relationship of the interaction between empagliflozin and NLRP3 inflammasome activation is still vague, which needs more attention in future, especially under a hyperglycemic background.

PPARs, belonging to nuclear receptors, are the key executives of regulating glucolipid metabolism [120]. Recently, new research elucidated that rosiglitazone, a PPAR-*γ* agonist, can generate an inhibitory effect on the activation of the NLRP3 inflammasome in mouse peritoneal macrophages exposed in LPS and nigericin [121]. However, it is unclear whether PPAR-*γ* can act on NLRP3 inflammasome in macrophages in the context of diabetic cardiomyopathy. Hence, there are gaps in the knowledge in this respect that need to be explored.

### 5.2. Phytochemicals

In recent years, some phytochemicals extracted from herbaceous plants that possess anti-inflammatory actions have attained much attention from scholars. Gypenosides (Gps), a bioactive compound extracted from Gynostemma pentaphylla, possesses multi-pharmacological properties. Gps inhibits NLRP3 inflammasome activation and reduces the production of IL-1*β* and IL-18, thereby alleviating myocardial injury in DCM animal models [122]. Other studies found that tilianin exerted a cardioprotective effect by reducing the associated proteins of pyroptosis, and this effect can be magnified by Syringin [123]. Coriolus versicolor (CV) is a mushroom with edible and medicinal value, which has an extract that can improve cardiac function in diabetic rats by reducing NF-κB expression and, subsequently, the NLRP3 inflammasome response [124]. The components of these compounds are complex, and their actions of targeting NLRP3 and pyroptosis still need to be further investigated.

Colchicine is an alkaloid derived from Liliaceae plants and is used primarily to treat autoinflammatory diseases, such as gout, familial Mediterranean fever, and acute or recurrent pericarditis. More recently, researchers found that colchicine can be used for therapy in coronary artery diseases [125,126]. Colchicine possesses an anti-inflammatory effect by inhibiting microtubule polymerization, the production of inflammatory mediators and adhesion molecule aggregation, and neutrophil migration [127]. Moreover, it has been revealed that colchicine can exert an anti-inflammatory effect by suppressing NLRP3 inflammasome-mediated IL-1*β* production [128]. As mentioned above, P2X7, a key factor for the NLRP3 inflammasome response to ATP, can be restrained by colchicine, resulting in a reduction in downstream molecule generation [129]. In light of the particular effect on NLRP3 inflammasome, colchicine may be a potential candidate for treating DCM, even though a relative study in this area is scarce.

### 5.3. Inhibiting Compounds

It is currently known that there have been several compounds that possess inhibitory effects on pyroptosis. CY-09, a special and directive inhibitor of NLRP3, has been shown to exert an inhibitory effect by combining directly into the ATP-binding motif of NACHT domain and restraining the ATPase activity of NLRP3 [130]. CY-09 can significantly improve metabolic disorders in rodent models of diabetics. Echoing a previous study, this finding again proved that the activity of ATPase is a key factor of NLRP3 oligomerization and activation [131]. Moreover, CY-09 can attenuate insulin resistance and hepatic steatosis by targeting NLRP3 in diabetic mice [132]. Consequently, CY-09 could be applied to the therapy for cardiovascular problems caused by diabetes in the future. However, additional studies regarding CY-09 acting on diabetic cardiomyopathy are a great necessity.

MCC950 is another effective small-molecular inhibitor of NLRP3, and it has been verified that this compound can exert a remarkable inhibitory effect on NLRP3 by disrupting ASC oligomerization [133]. It has been demonstrated that MCC950 can slow down the development of carotid artery plaques in Apolipoprotein E-deficient mice [134]. In addition, MCC950 improved the aortic plaque stability and vascular function in STZ-induced diabetic ApoE ^−/−^ mice and caused a reduction in NLRP3 and IL-1*β* protein levels. The same results were also found in in vitro tests, showing that MCC950 reduced the expression of NLRP3 downstream proteins in several cell lines, whether under high glucose or LPS conditions [135]. In acute myocardial infraction (AMI) mouse models, ^18F^-FDG PET image showed that MCC950 treatment decreased ^18F^-FDG inflammatory uptake and infiltration of the inflammatory cells, as well as the levels of NLRP3 and IL-1β [136]. The results mentioned above are undoubtedly inspiring; however, the impact of MCC950 in diabetic cardiomyopathy remains ill-informed. Hence, there is a large amount of work that needs to be completed before applicating in clinical practice.

Since the activation of GSDMD can directly trigger the occurrence of pyroptosis, targeting this crucial molecule may be a novel choice of treatment in DCM. Dimethyl fumarate (DMF), an intermediate product of the citric acid cycle, is regarded as a targeted inhibitor of GSDMD. DMF can respond to GSDMD at a pivotal cysteine residue to promote GSDMD succination, it then affects the interaction between GSDMD and caspases, and consequent reactions [137]. In rodent models, DMF can relieve familial Mediterranean fever as well as autoimmune encephalitis by inhibiting GSDMD. Disulfiram, a drug approved to treat alcoholism, was identified as a potent inhibitor of GSDMD by high-throughput biochemical screening [55]. The researchers found that disulfiram abolished the formation of the plasma membrane’s pore; however, the inhibiting actions of GSDMD and IL-1*β* were insignificant in cells and mice. Even so, it provides an attractive option for the treatment of inflammatory diseases, including DCM.

### 5.4. Non-Coding RNAs

In recent decades, the correlation of the non-coding RNAs(ncRNAs) to cardiovascular diseases has boasted significant attention. Among these, microRNAs(miRNAs) are the most-investigated class of ncRNAs. In DCM rat models, the expression of miRNA223 was clearly increased, and, after adding a miR223 inhibitor, cardiac fibrosis and the activation of NLRP3 inflammasome were notably relieved [138]. The same results were found in H9C2 cells treated with high glucose. Another study showed that miRNA9 can improve hyperglycemia-induced human ventricular cardiomyocyte injury by suppressing the activation of pyrotosis via the targeting of ELAVL1(ELAV-like protein1). In addition to that, the over-expression of miRNA30d can up-regulate the expression of key proteins of pyroptosis by targeting foxo3a and its downstream proteins, known as apoptosis repressors, with caspase (ARC) [139]. Based on the above, we can infer that miRNAs might be a fine choice for DCM through its inhibition of pyroptosis; however, there still remains much progress before its application in clinical practice.

Similarly, other types of ncRNAs also exert a remarkable effect on mediating NLRP3 or pyroptosis activation. LncRNA KCNQ1ot1 was increased in the left ventricular myocardium of DCM, and this gene silencing could suppress CFs pyroptosis and ameliorate the cardiac function and fibrosis [140]. Another study found that melatonin could relieve cardiac fibrosis and repress pyroptosis by inhibiting lncRNA MALAT1/miRNA-141-mediated pyroptosis [141]. Yang et al. reported that has_circ_0076631, also called caspase-1-associated circRNA (CACR), was elevated in the peripheral circulation of diabetic patients and HG pre-treated AC16 cells. CACR knockdown in cardiomyocytes can restrain the activation of caspase-1 induced by HG, which could also be regulated by miRNA-214-3p [142].

Altogether, ncRNAs may be a main factor of the mediation of pyroptosis activation, and this provides a prospective direction for the development of DCM medication. Despite a growing body of research focused on this field, an accurate explanation of the underlying mechanisms remains elusive. There is much progress left before we can consider translating these findings into clinical practice.

### 5.5. Protein Molecules

Proteins are vital components of living organisms. They play a part in regulating many biological processes, from cell growth to cell death, by forming specific complexes with DNA, as well as small ligands or other proteins. Visceral adipose-tissue-derived serine protease inhibitor (Vaspin), one kind of adipocytokines, can suppress NLRP3 inflammasome activation and the subsequent occurrence of pyroptosis by protecting autophagy in diabetic rats [143]. Furthermore, this inhibitory effect can be abrogated by an autophagy inhibitor. This means that Vaspin may be a good candidate for DCM treatment. Another study indicated that the anti-aging protein Klotho has distinct cardio-protective properties by restraining the NLRP3 inflammasome, either in in vivo or in vitro experiments [144]. Sirtuin 3 (SIRT3) is one of the class III histone deacetylases located in the mitochondria, which participates in multiple pathophysiological responses in the body. It has been revealed that SIRT3 silencing promoted the expression of NLRP3, caspase-1*p*20, and IL-1*β*, aggravating mitochondrial injury and necroptosis, which consequently deteriorated DCM in mice [145]. Beyond these findings, the Chemerin/CMKLR1 signaling pathway can promote pyroptosis by stimulating NLRP3 inflammasome activation and by worsening DCM development [134].

Are there other special substrates existing in interaction between pyroptosis and these proteins? How do the different protein folding patterns change in this process? Few studies have attempted to address these issues. Even so, studies concerning the interaction between pyroptosis and protein molecules provides a wider consideration for the development of therapy for diabetic cardiomyopathy.

## 6. Important Gaps in Knowledge in the Field

### 6.1. The Precise Relationship between Mitochondrial Dysfunction and NLRP3 Inflammasome

Mitochondria are dynamic organelles that perform functions other than energy production, such as maintaining calcium homeostasis and regulating cell proliferation and metabolism. It has been established that there is a strong link between mitochondria and NLRP3 inflammasome. Mitochondrial ROS can trigger the activation of NLRP3 inflammasome [38]. In addition, the single action of mitochondrial ROS is not sufficient to activate NLRP3 inflammasome; other factors need to be involved, such as mitochondrial membrane potential [40]. However, the precise relationship between them is still uncertain. Recent research has shown that the mitochondrial-localized adaptor protein ASC can promote the formation and activation of NLRP3 inflammasome through hyperacetylation. This indicates that there is a greater assembly of NLRP3 inflammasome on mitochondria [146]. However, the specific location of NLRP3 inflammasome assembly in mitochondria and whether it is related to other protein modification processes are yet to be investigated. Additionally, the causal relationship between NLRP3 inflammasome activation and mitochondrial dysfunction is unclear. It was previously thought that increased ROS production due to mitochondrial damage and Ca ^2+^-mediated mitochondrial damage can induce the activation of NLRP3 inflammasome [147]. However, there is also the contrary view that increased NLRP3 inflammasome activity can induce mitochondrial damage and the subsequent reduction in mtDNA copy number and ATP synthesis [148].

Therefore, the precise relationship between NLRP3 inflammasome and mitochondrial dysfunction needs further in-depth study.

### 6.2. The Exact Regulatory Mechanism of NLRP3 Inflammasome/pyroptosis Activation in Macrophages in the Context of Diabetic Cardiomyopathy

Cardiac-resident macrophages play a critical role in maintaining cardiac homeostasis through the uptake of subcellular vesicles containing damaged mitochondria and other waste products to prevent the accumulation of extracellular waste and the activation of NLRP3 inflammasome [149]. However, the differences in the mode of NLRP3 inflammasome activation between different subpopulations of cardiac-resident macrophages and peritoneal macrophages in the context of diabetes are unclear, this requires further investigation. Macrophage polarization towards pro-inflammatory M1 cells and anti-inflammatory M2 cells is essential for the maintenance of the host’s defenses. It has been reported that NLRP3 can regulate the transition of M1/M2 macrophages [150]. Ubiquitin-specific protease 19 (USP19) can regulate the activation of the NLRP3 inflammasome and prompt M2-like macrophage polarization by increasing autophagic flux and modulating the production of mitochondrial ROS [151]. This suggests that, in macrophages, the NLRP3 inflammasome activation has crosstalk signals with mitochondrial autophagy/autophagy and mitochondrial dysfunction. Nevertheless, the interaction between these signals is still elusive, especially in a diabetic heart.

Hence, further clarity is needed on these issues in the future.

## 7. Conclusions

Diabetic cardiomyopathy has been recognized for several decades, its incidence will continuously increase with the prevalence of diabetes. Against this grim backdrop, abundant investigations have been committed to explore potential pathogenesis mechanisms and to develop novel therapeutic approaches for DCM. Despite the production of several new drugs that target glycemic control and protect cardiovascular function, the morbidity of DCM does not show a downward trend. In preclinical studies, many underlying molecular mechanisms have been proposed, and some novel strategies have been confirmed to possess benefits on DCM animal models. This is undoubtedly encouraging news for researchers and clinicians, despite the precise mechanism remaining blurred and in need of further exploration. As cell death is the pivotal form of histopathological change in diabetic cardiomyopathy, the executors of the cell death pathway are clearly the therapeutic targets of DCM. As outlined above, pyroptosis is a new type of inflammatory PCD, which has been established in the genesis and development of DCM. Hence, blocking the pyroptosis signal pathway (or inflammasome activation) may have a clinical benefit in halting or delaying the progression of DCM.

To date, many interventions have been discovered to have the potential to inhibit pyroptosis, including traditional hypoglycemic agents, phytochemicals, specific inhibitory compounds, ncRNAs, and some special proteins. Among antidiabetics, metformin and SGLT2i have been the most extensively studied in relation to NLRP3 inflammasome/pyroptosis. The results of studies proved that these drugs are effective, but the precise molecular mechanism remains obscure. Additionally, phytochemicals and specific inhibitory compounds, also presented good results in the treatment of DCM by targeting NLRP3 inflammasome or pyroptosis in preclinical research. However, there are still many issues to be resolved before clinical translation can be applied, such as the exact pharmacological mechanism of these kinds of interventions and potential toxicities. Apart from these, ncRNAs and some special proteins also provide a novel insight for the research and development of new drugs for DCM.

Nevertheless, the details relating to the underlying molecular mechanisms of NLRP3 inflammasome/pyroptosis in diabetic heart diseases remain elusive. Moreover, the research of potential interventions for DCM targeting NLRP3 and pyroptosis is still in its infancy. For this reason, it is important to further explore the precise pathogenic link between NLRP3 inflammasome/pyroptosis and diabetic cardiomyopathy in the hope that this endeavor translates into promising therapeutic approaches for the sufferers of DCM.

## Abbreviations

ACR	apoptosis repressor with caspase
AMI	acute myocardial infarction
AGEs	advanced glycation end products
AIM2	absent in melanoma2-like receptors
AMPK	AMP-activated protein kinase
AS	atherosclerosis
ASC	apoptosis-associated speck-like protein containing
BTK	Bruton’s tyrosine kinase
CACR	caspase-1-associated circ RNA
CARD	caspase activation and recruitment domain
CFs	cardiac fibroblasts
CLRs	C-type lectin receptors
circ RNAs	circular RNAs
CMR	cardiac magnetic resonance
COX-2	Cyclooxygenase 2 (COX-2)
CV	coriolus versicolor
CVDs	cardiovascular diseases
DAMP	damage- associated molecular patterns
DCM	diabetic cardiomyopathy
DPP4	dipeptidyl peptidase4
ELAVL1	ELAV-like RNA-binding protein 1
GBP	guanylate-binding protein
Gps	Gypenosides
GSDMD	Gasdermin D
GSDMD-CT	Gasdermin C-terminal domain
GSDMD-NT	Gasdermin N-terminal domain
G3BP1	GTPase-activating protein (SH3 domain)-binding-protein 1
GV	Glucose variability
HF	heart failure
HG	high glucose
H/R	Hypoxia/Reoxygenation
IL-1*β*	Interleukin-1*β*
IL-18	Interleukin-18
INF-*γ*	Interferon-*γ*
LGE	late gadolinium enhancement
LPS	Lipopolysaccharide
LRR	Leucine-rich repeat
ncRNAs	non-coding RNAs
NEK7	NIMA-related kinase 7
NF-κB	nuclear factors -κB
NLRP3	NOD-like receptor pyrin domain containing 3
oxLDL	oxidized low-density lipoprotein
PAMP	pathogens-associated molecular patterns
PCD	programmed cell death
PRR	pattern recognition receptor
PPARs	peroxisome proliferator-activated receptors
P2X7	purinergic receptor P2X, ligand-gated ion channel 7
RAMI	recurrent acute myocardial infarction
RLRs	retinoic acid-inducible gene (RIG)-I-like receptors
ROS	reactive oxygen species
SGLT2	sodium-glucose cotransporter 2
SGLT2i	sodium-glucose cotransporter 2 inhibitor
SIRT3	sirtuin 3
STZ	streptozotocin
T2D	type 2 diabetes
TLRs	toll-like receptors
TRX	thioredoxin
TXNIP	thioredoxin-interacting protein
USP19	Ubiquitin-specific protease 19
Vaspin	visceral adipose tissue-derived serine protease inhibitor

## References

[1] De Simone G., Devereux R.B., Chinali M., Lee E.T., Galloway J.M., Barac A., Panza J.A., Howard B.V. (2010). Diabetes and incident heart failure in hypertensive and normotensive participants of the Strong Heart Study. J. Hypertens..

[2] Rubler S., Dlugash J., Yuceoglu Y.Z., Kumral T., Branwood A.W., Grishman A. (1972). New type of cardiomyopathy associated with diabetic glomerulosclerosis. Am. J. Cardiol..

[3] Maisch B., Alter P., Pankuweit S. (2011). Diabetic cardiomyopathy—Fact or fiction?. Herz.

[4] Tadic M., Cuspidi C., Calicchio F., Grassi G., Mancia G. (2020). Diabetic cardiomyopathy: How can cardiac magnetic resonance help?. Acta Diabetol..

[5] Kwong R.Y., Sattar H., Wu H., Vorobiof G., Gandla V., Steel K., Siu S., Brown K.A. (2008). Incidence and prognostic implication of unrecognized myocardial scar characterized by cardiac magnetic resonance in diabetic patients without clinical evidence of myocardial infarction. Circulation.

[6] Bugger H., Abel E.D. (2014). Molecular mechanisms of diabetic cardiomyopathy. Diabetologia.

[7] Zychlinsky A., Prevost M.C., Sansonetti P.J. (1992). Shigella flexneri induces apoptosis in infected macrophages. Nature.

[8] Cookson B.T., Brennan M.A. (2001). Pro-inflammatory programmed cell death. Trends Microbiol..

[9] Zhaolin Z., Jiaojiao C., Peng W., Yami L., Tingting Z., Jun T., Shiyuan W., Jinyan X., Dangheng W., Zhisheng J. (2019). OxLDL induces vascular endothelial cell pyroptosis through miR-125a-5p/TET2 pathway. J. Cell. Physiol..

[10] Wu P., Chen J., Chen J., Tao J., Wu S., Xu G., Wang Z., Wei D., Yin W. (2020). Trimethylamine N-oxide promotes apoE(−/−) mice atherosclerosis by inducing vascular endothelial cell pyroptosis via the SDHB/ROS pathway. J. Cell. Physiol..

[11] Luo B., Huang F., Liu Y., Liang Y., Wei Z., Ke H., Zeng Z., Huang W., He Y. (2017). NLRP3 Inflammasome as a Molecular Marker in Diabetic Cardiomyopathy. Front. Physiol..

[12] Shen S., He F., Cheng C., Xu B., Sheng J. (2021). Uric acid aggravates myocardial ischemia-reperfusion injury via ROS/NLRP3 pyroptosis pathway. Biomed. Pharm..

[13] Nazir S., Gadi I., Al-Dabet M.M., Elwakiel A., Kohli S., Ghosh S., Manoharan J., Ranjan S., Bock F., Braun-Dullaeus R.C. (2017). Cytoprotective activated protein C averts Nlrp3 inflammasome-induced ischemia-reperfusion injury via mTORC1 inhibition. Blood.

[14] Li W., Chen L., Xiao Y. (2020). Apigenin protects against ischemia-/hypoxia-induced myocardial injury by mediating pyroptosis and apoptosis. Vitr. Cell. Dev. Biol. Anim..

[15] Man S.M., Karki R., Kanneganti T.D. (2017). Molecular mechanisms and functions of pyroptosis, inflammatory caspases and inflammasomes in infectious diseases. Immunol. Rev..

[16] Gavrilin M.A., Abdelaziz D.H., Mostafa M., Abdulrahman B.A., Grandhi J., Akhter A., Abu Khweek A., Aubert D.F., Valvano M.A., Wewers M.D. (2012). Activation of the pyrin inflammasome by intracellular Burkholderia cenocepacia. J. Immunol..

[17] Hornung V., Ablasser A., Charrel-Dennis M., Bauernfeind F., Horvath G., Caffrey D.R., Latz E., Fitzgerald K.A. (2009). AIM2 recognizes cytosolic dsDNA and forms a caspase-1-activating inflammasome with ASC. Nature.

[18] Awad F., Assrawi E., Louvrier C., Jumeau C., Georgin-Lavialle S., Grateau G., Amselem S., Giurgea I., Karabina S.A. (2018). Inflammasome biology, molecular pathology and therapeutic implications. Pharm. Ther..

[19] Wen H., Miao E.A., Ting J.P. (2013). Mechanisms of NOD-like receptor-associated inflammasome activation. Immunity.

[20] Latz E., Xiao T.S., Stutz A. (2013). Activation and regulation of the inflammasomes. Nat. Rev. Immunol..

[21] Boucher D., Monteleone M., Coll R.C., Chen K.W., Ross C.M., Teo J.L., Gomez G.A., Holley C.L., Bierschenk D., Stacey K.J. (2018). Caspase-1 self-cleavage is an intrinsic mechanism to terminate inflammasome activity. J. Exp. Med..

[22] Denes A., Lopez-Castejon G., Brough D. (2012). Caspase-1: Is IL-1 just the tip of the ICEberg?. Cell Death Dis..

[23] Vande Walle L., Lamkanfi M. (2020). Snapshot of a Deadly Embrace: The Caspase-1-GSDMD Interface. Immunity.

[24] Wang K., Sun Q., Zhong X., Zeng M., Zeng H., Shi X., Li Z., Wang Y., Zhao Q., Shao F. (2020). Structural Mechanism for GSDMD Targeting by Autoprocessed Caspases in Pyroptosis. Cell.

[25] Guo H., Callaway J.B., Ting J.P. (2015). Inflammasomes: Mechanism of action, role in disease, and therapeutics. Nat. Med..

[26] Chen S., Sun B. (2013). Negative regulation of NLRP3 inflammasome signaling. Protein Cell.

[27] Huang Y., Xu W., Zhou R. (2021). NLRP3 inflammasome activation and cell death. Cell. Mol. Immunol..

[28] Gram A.M., Frenkel J., Ressing M.E. (2012). Inflammasomes and viruses: Cellular defence versus viral offence. J. Gen. Viro.l.

[29] Mariathasan S., Weiss D.S., Newton K., McBride J., O’Rourke K., Roose-Girma M., Lee W.P., Weinrauch Y., Monack D.M., Dixit V.M. (2006). Cryopyrin activates the inflammasome in response to toxins and ATP. Nature.

[30] Wen H., Gris D., Lei Y., Jha S., Zhang L., Huang M.T., Brickey W.J., Ting J.P. (2011). Fatty acid-induced NLRP3-ASC inflammasome activation interferes with insulin signaling. Nat. Immunol..

[31] Dostert C., Petrilli V., Van Bruggen R., Steele C., Mossman B.T., Tschopp J. (2008). Innate immune activation through Nalp3 inflammasome sensing of asbestos and silica. Science.

[32] Di Virgilio F., Dal Ben D., Sarti A.C., Giuliani A.L., Falzoni S. (2017). The P2X7 Receptor in Infection and Inflammation. Immunity.

[33] Munoz-Planillo R., Kuffa P., Martinez-Colon G., Smith B.L., Rajendiran T.M., Nunez G. (2013). K(+) efflux is the common trigger of NLRP3 inflammasome activation by bacterial toxins and particulate matter. Immunity.

[34] Duewell P., Kono H., Rayner K.J., Sirois C.M., Vladimer G., Bauernfeind F.G., Abela G.S., Franchi L., Nunez G., Schnurr M. (2010). NLRP3 inflammasomes are required for atherogenesis and activated by cholesterol crystals. Nature.

[35] Hornung V., Bauernfeind F., Halle A., Samstad E.O., Kono H., Rock K.L., Fitzgerald K.A., Latz E. (2008). Silica crystals and aluminum salts activate the NALP3 inflammasome through phagosomal destabilization. Nat. Immunol..

[36] Hoseini Z., Sepahvand F., Rashidi B., Sahebkar A., Masoudifar A., Mirzaei H. (2018). NLRP3 inflammasome: Its regulation and involvement in atherosclerosis. J. Cell. Physiol..

[37] Yang Y., Wang H., Kouadir M., Song H., Shi F. (2019). Recent advances in the mechanisms of NLRP3 inflammasome activation and its inhibitors. Cell Death Dis..

[38] Zhou R., Yazdi A.S., Menu P., Tschopp J. (2011). A role for mitochondria in NLRP3 inflammasome activation. Nature.

[39] Abais J.M., Xia M., Zhang Y., Boini K.M., Li P.L. (2015). Redox regulation of NLRP3 inflammasomes: ROS as trigger or effector?. Antioxid. Redox Signal..

[40] Ichinohe T., Yamazaki T., Koshiba T., Yanagi Y. (2013). Mitochondrial protein mitofusin 2 is required for NLRP3 inflammasome activation after RNA virus infection. Proc. Natl. Acad. Sci. USA.

[41] Kandli M., Feige E., Chen A., Kilfin G., Motro B. (2000). Isolation and characterization of two evolutionarily conserved murine kinases (Nek6 and nek7) related to the fungal mitotic regulator, NIMA. Genomics.

[42] Petrilli V., Papin S., Dostert C., Mayor A., Martinon F., Tschopp J. (2007). Activation of the NALP3 inflammasome is triggered by low intracellular potassium concentration. Cell Death Differ..

[43] He Y., Zeng M.Y., Yang D., Motro B., Nunez G. (2016). NEK7 is an essential mediator of NLRP3 activation downstream of potassium efflux. Nature.

[44] Fischer U., Janicke R.U., Schulze-Osthoff K. (2003). Many cuts to ruin: A comprehensive update of caspase substrates. Cell Death Differ..

[45] Martinon F., Tschopp J. (2004). Inflammatory caspases: Linking an intracellular innate immune system to autoinflammatory diseases. Cell.

[46] Zhang Y., Chen X., Gueydan C., Han J. (2018). Plasma membrane changes during programmed cell deaths. Cell Res..

[47] Zhaolin Z., Guohua L., Shiyuan W., Zuo W. (2019). Role of pyroptosis in cardiovascular disease. Cell Prolif..

[48] de Vasconcelos N.M., Lamkanfi M. (2020). Recent Insights on Inflammasomes, Gasdermin Pores, and Pyroptosis. Cold Spring Harb. Perspect. Biol..

[49] Miao E.A., Rajan J.V., Aderem A. (2011). Caspase-1-induced pyroptotic cell death. Immunol. Rev..

[50] Dinarello C.A. (2009). Interleukin-1beta and the autoinflammatory diseases. N. Engl. J. Med..

[51] Wilson K.P., Black J.A., Thomson J.A., Kim E.E., Griffith J.P., Navia M.A., Murcko M.A., Chambers S.P., Aldape R.A., Raybuck S.A. (1994). Structure and mechanism of interleukin-1 beta converting enzyme. Nature.

[52] Ding J., Wang K., Liu W., She Y., Sun Q., Shi J., Sun H., Wang D.C., Shao F. (2016). Pore-forming activity and structural autoinhibition of the gasdermin family. Nature.

[53] Liu X., Zhang Z., Ruan J., Pan Y., Magupalli V.G., Wu H., Lieberman J. (2016). Inflammasome-activated gasdermin D causes pyroptosis by forming membrane pores. Nature.

[54] Liu Z., Wang C., Yang J., Zhou B., Yang R., Ramachandran R., Abbott D.W., Xiao T.S. (2019). Crystal Structures of the Full-Length Murine and Human Gasdermin D Reveal Mechanisms of Autoinhibition, Lipid Binding, and Oligomerization. Immunity.

[55] Hu J.J., Liu X., Xia S., Zhang Z., Zhang Y., Zhao J., Ruan J., Luo X., Lou X., Bai Y. (2020). FDA-approved disulfiram inhibits pyroptosis by blocking gasdermin D pore formation. Nat. Immunol..

[56] Russo H.M., Rathkey J., Boyd-Tressler A., Katsnelson M.A., Abbott D.W., Dubyak G.R. (2016). Active Caspase-1 Induces Plasma Membrane Pores That Precede Pyroptotic Lysis and Are Blocked by Lanthanides. J. Immunol..

[57] de Vasconcelos N.M., Van Opdenbosch N., Van Gorp H., Parthoens E., Lamkanfi M. (2019). Single-cell analysis of pyroptosis dynamics reveals conserved GSDMD-mediated subcellular events that precede plasma membrane rupture. Cell Death Differ..

[58] Akhter A., Caution K., Abu Khweek A., Tazi M., Abdulrahman B.A., Abdelaziz D.H., Voss O.H., Doseff A.I., Hassan H., Azad A.K. (2012). Caspase-11 promotes the fusion of phagosomes harboring pathogenic bacteria with lysosomes by modulating actin polymerization. Immunity.

[59] Knodler L.A., Crowley S.M., Sham H.P., Yang H., Wrande M., Ma C., Ernst R.K., Steele-Mortimer O., Celli J., Vallance B.A. (2014). Noncanonical inflammasome activation of caspase-4/caspase-11 mediates epithelial defenses against enteric bacterial pathogens. Cell Host Microbe.

[60] Wang X., Quinn P.J. (2010). Lipopolysaccharide: Biosynthetic pathway and structure modification. Prog. Lipid Res..

[61] Santos J.C., Dick M.S., Lagrange B., Degrandi D., Pfeffer K., Yamamoto M., Meunier E., Pelczar P., Henry T., Broz P. (2018). LPS targets host guanylate-binding proteins to the bacterial outer membrane for non-canonical inflammasome activation. EMBO J..

[62] Kayagaki N., Warming S., Lamkanfi M., Vande Walle L., Louie S., Dong J., Newton K., Qu Y., Liu J., Heldens S. (2011). Non-canonical inflammasome activation targets caspase-11. Nature.

[63] Shi J., Zhao Y., Wang K., Shi X., Wang Y., Huang H., Zhuang Y., Cai T., Wang F., Shao F. (2015). Cleavage of GSDMD by inflammatory caspases determines pyroptotic cell death. Nature.

[64] Kayagaki N., Stowe I.B., Lee B.L., O’Rourke K., Anderson K., Warming S., Cuellar T., Haley B., Roose-Girma M., Phung Q.T. (2015). Caspase-11 cleaves gasdermin D for non-canonical inflammasome signalling. Nature.

[65] Chen K.W., Demarco B., Heilig R., Shkarina K., Boettcher A., Farady C.J., Pelczar P., Broz P. (2019). Extrinsic and intrinsic apoptosis activate pannexin-1 to drive NLRP3 inflammasome assembly. EMBO J..

[66] Jia G., DeMarco V.G., Sowers J.R. (2016). Insulin resistance and hyperinsulinaemia in diabetic cardiomyopathy. Nat. Rev. Endocrinol..

[67] Jia G., Hill M.A., Sowers J.R. (2018). Diabetic Cardiomyopathy: An Update of Mechanisms Contributing to This Clinical Entity. Circ. Res..

[68] Devereux R.B., Roman M.J., Paranicas M., O’Grady M.J., Lee E.T., Welty T.K., Fabsitz R.R., Robbins D., Rhoades E.R., Howard B.V. (2000). Impact of diabetes on cardiac structure and function: The strong heart study. Circulation.

[69] Shimizu M., Umeda K., Sugihara N., Yoshio H., Ino H., Takeda R., Okada Y., Nakanishi I. (1993). Collagen remodelling in myocardia of patients with diabetes. J. Clin. Pathol..

[70] Goldin A., Beckman J.A., Schmidt A.M., Creager M.A. (2006). Advanced glycation end products: Sparking the development of diabetic vascular injury. Circulation.

[71] Huynh K., Kiriazis H., Du X.J., Love J.E., Gray S.P., Jandeleit-Dahm K.A., McMullen J.R., Ritchie R.H. (2013). Targeting the upregulation of reactive oxygen species subsequent to hyperglycemia prevents type 1 diabetic cardiomyopathy in mice. Free Radic. Biol. Med..

[72] Wilson A.J., Gill E.K., Abudalo R.A., Edgar K.S., Watson C.J., Grieve D.J. (2018). Reactive oxygen species signalling in the diabetic heart: Emerging prospect for therapeutic targeting. Heart.

[73] Montaigne D., Marechal X., Coisne A., Debry N., Modine T., Fayad G., Potelle C., El Arid J.M., Mouton S., Sebti Y. (2014). Myocardial contractile dysfunction is associated with impaired mitochondrial function and dynamics in type 2 diabetic but not in obese patients. Circulation.

[74] Chen Y., Hua Y., Li X., Arslan I.M., Zhang W., Meng G. (2020). Distinct Types of Cell Death and the Implication in Diabetic Cardiomyopathy. Front Pharm..

[75] Parim B., Sathibabu Uddandrao V.V., Saravanan G. (2018). Diabetic cardiomyopathy: Molecular mechanisms, detrimental effects of conventional treatment, and beneficial effects of natural therapy. Heart Fail. Rev..

[76] Bolivar B.E., Vogel T.P., Bouchier-Hayes L. (2019). Inflammatory caspase regulation: Maintaining balance between inflammation and cell death in health and disease. FEBS J..

[77] Zeng C., Wang R., Tan H. (2019). Role of Pyroptosis in Cardiovascular Diseases and its Therapeutic Implications. Int. J. Biol. Sci..

[78] Luo B., Li B., Wang W., Liu X., Xia Y., Zhang C., Zhang M., Zhang Y., An F. (2014). NLRP3 gene silencing ameliorates diabetic cardiomyopathy in a type 2 diabetes rat model. PLoS ONE.

[79] Greten F.R., Arkan M.C., Bollrath J., Hsu L.C., Goode J., Miething C., Goktuna S.I., Neuenhahn M., Fierer J., Paxian S. (2007). NF-kappaB is a negative regulator of IL-1beta secretion as revealed by genetic and pharmacological inhibition of IKKbeta. Cell.

[80] Zhou R., Tardivel A., Thorens B., Choi I., Tschopp J. (2010). Thioredoxin-interacting protein links oxidative stress to inflammasome activation. Nat. Immunol..

[81] Szpigel A., Hainault I., Carlier A., Venteclef N., Batto A.-F., Hajduch E., Bernard C., Ktorza A., Gautier J.-F., Ferré P. (2017). Lipid environment induces ER stress, TXNIP expression and inflammation in immune cells of individuals with type 2 diabetes. Diabetologia.

[82] Hutchinson K.R., Lord C.K., West T.A., Stewart J.A. (2013). Cardiac fibroblast-dependent extracellular matrix accumulation is associated with diastolic stiffness in type 2 diabetes. PLoS ONE.

[83] Goldsmith E.C., Bradshaw A.D., Zile M.R., Spinale F.G. (2014). Myocardial fibroblast-matrix interactions and potential therapeutic targets. J. Mol. Cell. Cardiol..

[84] Kawaguchi M., Takahashi M., Hata T., Kashima Y., Usui F., Morimoto H., Izawa A., Takahashi Y., Masumoto J., Koyama J. (2011). Inflammasome activation of cardiac fibroblasts is essential for myocardial ischemia/reperfusion injury. Circulation.

[85] Shi P., Zhao X.D., Shi K.H., Ding X.S., Tao H. (2021). MiR-21-3p triggers cardiac fibroblasts pyroptosis in diabetic cardiac fibrosis via inhibiting androgen receptor. Exp. Cell Res..

[86] Qiu Z., Lei S., Zhao B., Wu Y., Su W., Liu M., Meng Q., Zhou B., Leng Y., Xia Z.Y. (2017). NLRP3 Inflammasome Activation-Mediated Pyroptosis Aggravates Myocardial Ischemia/Reperfusion Injury in Diabetic Rats. Oxid. Med. Cell. Longev..

[87] Qiu Z., He Y., Ming H., Lei S., Leng Y., Xia Z.Y. (2019). Lipopolysaccharide (LPS) Aggravates High Glucose- and Hypoxia/Reoxygenation-Induced Injury through Activating ROS-Dependent NLRP3 Inflammasome-Mediated Pyroptosis in H9C2 Cardiomyocytes. J. Diabetes Res..

[88] Bardini G., Dicembrini I., Cresci B., Rotella C.M. (2010). Inflammation markers and metabolic characteristics of subjects with 1-h plasma glucose levels. Diabetes Care.

[89] Muller S., Martin S., Koenig W., Hanifi-Moghaddam P., Rathmann W., Haastert B., Giani G., Illig T., Thorand B., Kolb H. (2002). Impaired glucose tolerance is associated with increased serum concentrations of interleukin 6 and co-regulated acute-phase proteins but not TNF-alpha or its receptors. Diabetologia.

[90] Urbina P., Singla D.K. (2014). BMP-7 attenuates adverse cardiac remodeling mediated through M2 macrophages in prediabetic cardiomyopathy. Am. J. Physiol. Heart Circ. Physiol..

[91] Russo I., Frangogiannis N.G. (2016). Diabetes-associated cardiac fibrosis: Cellular effectors, molecular mechanisms and therapeutic opportunities. J. Mol. Cell. Cardiol..

[92] Lin H.B., Wei G.S., Li F.X., Guo W.J., Hong P., Weng Y.Q., Zhang Q.Q., Xu S.Y., Liang W.B., You Z.J. (2020). Macrophage-NLRP3 Inflammasome Activation Exacerbates Cardiac Dysfunction after Ischemic Stroke in a Mouse Model of Diabetes. Neurosci. Bull..

[93] Pal Singh S., Dammeijer F., Hendriks R.W. (2018). Role of Bruton’s tyrosine kinase in B cells and malignancies. Mol. Cancer.

[94] Purvis G.S.D., Collino M., Aranda-Tavio H., Chiazza F., O’Riordan C.E., Zeboudj L., Mohammad S., Collotta D., Verta R., Guisot N.E.S. (2020). Inhibition of Bruton’s TK regulates macrophage NF-kappaB and NLRP3 inflammasome activation in metabolic inflammation. Br. J. Pharm..

[95] Madonna R., De Caterina R. (2011). Cellular and molecular mechanisms of vascular injury in diabetes—Part II: Cellular mechanisms and therapeutic targets. Vasc. Pharm..

[96] Correa R., Silva L.F.F., Ribeiro D.J.S., Almeida R.D.N., Santos I.O., Correa L.H., de Sant’Ana L.P., Assuncao L.S., Bozza P.T., Magalhaes K.G. (2019). Lysophosphatidylcholine Induces NLRP3 Inflammasome-Mediated Foam Cell Formation and Pyroptosis in Human Monocytes and Endothelial Cells. Front. Immunol..

[97] Zeng Z., Zheng Q., Chen J., Tan X., Li Q., Ding L., Zhang R., Lin X. (2020). FGF21 mitigates atherosclerosis via inhibition of NLRP3 inflammasome-mediated vascular endothelial cells pyroptosis. Exp. Cell Res..

[98] Burleigh M.E., Babaev V.R., Yancey P.G., Major A.S., McCaleb J.L., Oates J.A., Morrow J.D., Fazio S., Linton M.F. (2005). Cyclooxygenase-2 promotes early atherosclerotic lesion formation in ApoE-deficient and C57BL/6 mice. J. Mol. Cell. Cardiol..

[99] Hua K.F., Chou J.C., Ka S.M., Tasi Y.L., Chen A., Wu S.H., Chiu H.W., Wong W.T., Wang Y.F., Tsai C.L. (2015). Cyclooxygenase-2 regulates NLRP3 inflammasome-derived IL-1beta production. J. Cell. Physiol..

[100] Rajamaki K., Lappalainen J., Oorni K., Valimaki E., Matikainen S., Kovanen P.T., Eklund K.K. (2010). Cholesterol crystals activate the NLRP3 inflammasome in human macrophages: A novel link between cholesterol metabolism and inflammation. PLoS ONE.

[101] Jacoby R.M., Nesto R.W. (1992). Acute myocardial infarction in the diabetic patient: Pathophysiology, clinical course and prognosis. J. Am. Coll. Cardiol..

[102] van Hout G.P., Bosch L., Ellenbroek G.H., de Haan J.J., van Solinge W.W., Cooper M.A., Arslan F., de Jager S.C., Robertson A.A., Pasterkamp G. (2017). The selective NLRP3-inflammasome inhibitor MCC950 reduces infarct size and preserves cardiac function in a pig model of myocardial infarction. Eur. Heart J..

[103] Meng Z., Song M.Y., Li C.F., Zhao J.Q. (2017). shRNA interference of NLRP3 inflammasome alleviate ischemia reperfusion-induced myocardial damage through autophagy activation. Biochem. Biophys. Res. Commun..

[104] Ceriello A., Monnier L., Owens D. (2019). Glycaemic variability in diabetes: Clinical and therapeutic implications. Lancet Diabetes Endocrinol..

[105] Xia J., Zhang J., Chang J., Tian Y., Li J., Zhang B., Zeng X., Yin C. (2020). The effects of glycaemic variability on intimal hyperplasia and plaque stability after stenting via autophagy-mediated G3BP1/NLRP3 inflammasome. Ann Transl Med.

[106] Flory J., Lipska K. (2019). Metformin in 2019. JAMA.

[107] Yang F., Qin Y., Wang Y., Meng S., Xian H., Che H., Lv J., Li Y., Yu Y., Bai Y. (2019). Metformin Inhibits the NLRP3 Inflammasome via AMPK/mTOR-dependent Effects in Diabetic Cardiomyopathy. Int. J. Biol. Sci..

[108] Tang G., Duan F., Li W., Wang Y., Zeng C., Hu J., Li H., Zhang X., Chen Y., Tan H. (2019). Metformin inhibited Nod-like receptor protein 3 inflammasomes activation and suppressed diabetes-accelerated atherosclerosis in apoE(−/−) mice. Biomed. Pharm..

[109] Fei Q., Ma H., Zou J., Wang W., Zhu L., Deng H., Meng M., Tan S., Zhang H., Xiao X. (2020). Metformin protects against ischaemic myocardial injury by alleviating autophagy-ROS-NLRP3-mediated inflammatory response in macrophages. J. Mol. Cell. Cardiol..

[110] Ferrannini E. (2017). Sodium-Glucose Co-transporters and Their Inhibition: Clinical Physiology. Cell Metab..

[111] McMurray J.J.V., Solomon S.D., Inzucchi S.E., Kober L., Kosiborod M.N., Martinez F.A., Ponikowski P., Sabatine M.S., Anand I.S., Belohlavek J. (2019). Dapagliflozin in Patients with Heart Failure and Reduced Ejection Fraction. N. Engl. J. Med..

[112] Kosiborod M.N., Jhund P.S., Docherty K.F., Diez M., Petrie M.C., Verma S., Nicolau J.C., Merkely B., Kitakaze M., DeMets D.L. (2020). Effects of Dapagliflozin on Symptoms, Function, and Quality of Life in Patients With Heart Failure and Reduced Ejection Fraction: Results From the DAPA-HF Trial. Circulation.

[113] Brown A.J.M., Gandy S., McCrimmon R., Houston J.G., Struthers A.D., Lang C.C. (2020). A randomized controlled trial of dapagliflozin on left ventricular hypertrophy in people with type two diabetes: The DAPA-LVH trial. Eur. Heart J..

[114] Chen H., Tran D., Yang H.C., Nylander S., Birnbaum Y., Ye Y. (2020). Dapagliflozin and Ticagrelor Have Additive Effects on the Attenuation of the Activation of the NLRP3 Inflammasome and the Progression of Diabetic Cardiomyopathy: An AMPK-mTOR Interplay. Cardiovasc. Drugs Ther..

[115] Ye Y., Bajaj M., Yang H.C., Perez-Polo J.R., Birnbaum Y. (2017). SGLT-2 Inhibition with Dapagliflozin Reduces the Activation of the Nlrp3/ASC Inflammasome and Attenuates the Development of Diabetic Cardiomyopathy in Mice with Type 2 Diabetes. Further Augmentation of the Effects with Saxagliptin, a DPP4 Inhibitor. Cardiovasc. Drugs Ther..

[116] Zinman B., Wanner C., Lachin J.M., Fitchett D., Bluhmki E., Hantel S., Mattheus M., Devins T., Johansen O.E., Woerle H.J. (2015). Empagliflozin, Cardiovascular Outcomes, and Mortality in Type 2 Diabetes. N. Engl. J. Med..

[117] Byrne N.J., Matsumura N., Maayah Z.H., Ferdaoussi M., Takahara S., Darwesh A.M., Levasseur J.L., Jahng J.W.S., Vos D., Parajuli N. (2020). Empagliflozin Blunts Worsening Cardiac Dysfunction Associated With Reduced NLRP3 (Nucleotide-Binding Domain-Like Receptor Protein 3) Inflammasome Activation in Heart Failure. Circ. Heart Fail..

[118] Quagliariello V., De Laurentiis M., Rea D., Barbieri A., Monti M.G., Carbone A., Paccone A., Altucci L., Conte M., Canale M.L. (2021). The SGLT-2 inhibitor empagliflozin improves myocardial strain, reduces cardiac fibrosis and pro-inflammatory cytokines in non-diabetic mice treated with doxorubicin. Cardiovasc. Diabetol..

[119] Gordon M., Meagher P., Connelly K.A. (2021). Effect of Empagliflozin and Liraglutide on the Nucleotide-Binding and Oligomerization Domain-Like Receptor Family Pyrin Domain-Containing 3 Inflammasome in a Rodent Model of Type 2 Diabetes Mellitus. Can. J. Diabetes.

[120] Lee T.W., Bai K.J., Lee T.I., Chao T.F., Kao Y.H., Chen Y.J. (2017). PPARs modulate cardiac metabolism and mitochondrial function in diabetes. J. Biomed. Sci..

[121] Yang C.-C., Wu C.-H., Lin T.-C., Cheng Y.-N., Chang C.-S., Lee K.-T., Tsai P.-J., Tsai Y.-S. (2021). Inhibitory effect of PPARγ on NLRP3 inflammasome activation. Theranostics.

[122] Zhang H., Chen X., Zong B., Yuan H., Wang Z., Wei Y., Wang X., Liu G., Zhang J., Li S. (2018). Gypenosides improve diabetic cardiomyopathy by inhibiting ROS-mediated NLRP3 inflammasome activation. J. Cell. Mol. Med..

[123] Yao J., Li Y., Jin Y., Chen Y., Tian L., He W. (2021). Synergistic cardioptotection by tilianin and syringin in diabetic cardiomyopathy involves interaction of TLR4/NF-kappaB/NLRP3 and PGC1a/SIRT3 pathways. Int. Immunopharmacol..

[124] Wang Y., Li H., Li Y., Zhao Y., Xiong F., Liu Y., Xue H., Yang Z., Ni S., Sahil A. (2019). Coriolus versicolor alleviates diabetic cardiomyopathy by inhibiting cardiac fibrosis and NLRP3 inflammasome activation. Phytother. Res..

[125] Nidorf S.M., Fiolet A.T.L., Mosterd A., Eikelboom J.W., Schut A., Opstal T.S.J., The S.H.K., Xu X.F., Ireland M.A., Lenderink T. (2020). Colchicine in Patients with Chronic Coronary Disease. N. Engl. J. Med..

[126] Tong D.C., Quinn S., Nasis A., Hiew C., Roberts-Thomson P., Adams H., Sriamareswaran R., Htun N.M., Wilson W., Stub D. (2020). Colchicine in Patients With Acute Coronary Syndrome: The Australian COPS Randomized Clinical Trial. Circulation.

[127] Martinez G.J., Celermajer D.S., Patel S. (2018). The NLRP3 inflammasome and the emerging role of colchicine to inhibit atherosclerosis-associated inflammation. Atherosclerosis.

[128] Martinon F., Petrilli V., Mayor A., Tardivel A., Tschopp J. (2006). Gout-associated uric acid crystals activate the NALP3 inflammasome. Nature.

[129] Marques-da-Silva C., Chaves M.M., Castro N.G., Coutinho-Silva R., Guimaraes M.Z. (2011). Colchicine inhibits cationic dye uptake induced by ATP in P2X2 and P2X7 receptor-expressing cells: Implications for its therapeutic action. Br. J. Pharm..

[130] Jiang H., He H., Chen Y., Huang W., Cheng J., Ye J., Wang A., Tao J., Wang C., Liu Q. (2017). Identification of a selective and direct NLRP3 inhibitor to treat inflammatory disorders. J. Exp. Med..

[131] Duncan J.A., Bergstralh D.T., Wang Y., Willingham S.B., Ye Z., Zimmermann A.G., Ting J.P. (2007). Cryopyrin/NALP3 binds ATP/dATP, is an ATPase, and requires ATP binding to mediate inflammatory signaling. Proc. Natl. Acad. Sci. USA.

[132] Wang X., Sun K., Zhou Y., Wang H., Zhou Y., Liu S., Nie Y., Li Y. (2021). NLRP3 inflammasome inhibitor CY-09 reduces hepatic steatosis in experimental NAFLD mice. Biochem. Biophys. Res. Commun..

[133] Coll R.C., Robertson A.A., Chae J.J., Higgins S.C., Munoz-Planillo R., Inserra M.C., Vetter I., Dungan L.S., Monks B.G., Stutz A. (2015). A small-molecule inhibitor of the NLRP3 inflammasome for the treatment of inflammatory diseases. Nat. Med..

[134] van der Heijden T., Kritikou E., Venema W., van Duijn J., van Santbrink P.J., Slutter B., Foks A.C., Bot I., Kuiper J. (2017). NLRP3 Inflammasome Inhibition by MCC950 Reduces Atherosclerotic Lesion Development in Apolipoprotein E-Deficient Mice-Brief Report. Arter. Thromb. Vasc. Biol..

[135] Sharma A., Choi J.S.Y., Stefanovic N., Al-Sharea A., Simpson D.S., Mukhamedova N., Jandeleit-Dahm K., Murphy A.J., Sviridov D., Vince J.E. (2021). Specific NLRP3 Inhibition Protects Against Diabetes-Associated Atherosclerosis. Diabetes.

[136] Li X., Yang W., Ma W., Zhou X., Quan Z., Li G., Liu D., Zhang Q., Han D., Gao B. (2021). (18)F-FDG PET imaging-monitored anti-inflammatory therapy for acute myocardial infarction: Exploring the role of MCC950 in murine model. J. Nucl. Cardiol..

[137] Humphries F., Shmuel-Galia L., Ketelut-Carneiro N., Li S., Wang B., Nemmara V.V., Wilson R., Jiang Z., Khalighinejad F., Muneeruddin K. (2020). Succination inactivates gasdermin D and blocks pyroptosis. Science.

[138] Xu D., Zhang X., Chen X., Yang S., Chen H. (2020). Inhibition of miR-223 attenuates the NLRP3 inflammasome activation, fibrosis, and apoptosis in diabetic cardiomyopathy. Life Sci..

[139] Li X., Du N., Zhang Q., Li J., Chen X., Liu X., Hu Y., Qin W., Shen N., Xu C. (2014). MicroRNA-30d regulates cardiomyocyte pyroptosis by directly targeting foxo3a in diabetic cardiomyopathy. Cell Death Dis..

[140] Yang F., Qin Y., Lv J., Wang Y., Che H., Chen X., Jiang Y., Li A., Sun X., Yue E. (2018). Silencing long non-coding RNA Kcnq1ot1 alleviates pyroptosis and fibrosis in diabetic cardiomyopathy. Cell Death Dis..

[141] Che H., Wang Y., Li H., Li Y., Sahil A., Lv J., Liu Y., Yang Z., Dong R., Xue H. (2020). Melatonin alleviates cardiac fibrosis via inhibiting lncRNA MALAT1/miR-141-mediated NLRP3 inflammasome and TGF-beta1/Smads signaling in diabetic cardiomyopathy. FASEB J..

[142] Yang F., Li A., Qin Y., Che H., Wang Y., Lv J., Li Y., Li H., Yue E., Ding X. (2019). A Novel Circular RNA Mediates Pyroptosis of Diabetic Cardiomyopathy by Functioning as a Competing Endogenous RNA. Mol. Ther. Nucleic Acids.

[143] Li X., Ke X., Li Z., Li B. (2019). Vaspin prevents myocardial injury in rats model of diabetic cardiomyopathy by enhancing autophagy and inhibiting inflammation. Biochem. Biophys. Res. Commun..

[144] Li X., Li Z., Li B., Zhu X., Lai X. (2019). Klotho improves diabetic cardiomyopathy by suppressing the NLRP3 inflammasome pathway. Life Sci..

[145] Song S., Ding Y., Dai G.L., Zhang Y., Xu M.T., Shen J.R., Chen T.T., Chen Y., Meng G.L. (2021). Sirtuin 3 deficiency exacerbates diabetic cardiomyopathy via necroptosis enhancement and NLRP3 activation. Acta Pharmacol. Sin..

[146] Deng Y., Xie M., Li Q., Xu X., Ou W., Zhang Y., Xiao H., Yu H., Zheng Y., Liang Y. (2021). Targeting Mitochondria-Inflammation Circuit by beta-Hydroxybutyrate Mitigates HFpEF. Circ. Res..

[147] Murakami T., Ockinger J., Yu J., Byles V., McColl A., Hofer A.M., Horng T. (2012). Critical role for calcium mobilization in activation of the NLRP3 inflammasome. Proc. Natl. Acad. Sci. USA.

[148] Chen Y., Zeng M., Zhang Y., Guo H., Ding W., Sun T. (2021). Nlrp3 Deficiency Alleviates Angiotensin II-Induced Cardiomyopathy by Inhibiting Mitochondrial Dysfunction. Oxidative Med. Cell. Longev..

[149] Nicolas-Avila J.A., Lechuga-Vieco A.V., Esteban-Martinez L., Sanchez-Diaz M., Diaz-Garcia E., Santiago D.J., Rubio-Ponce A., Li J.L., Balachander A., Quintana J.A. (2020). A Network of Macrophages Supports Mitochondrial Homeostasis in the Heart. Cell.

[150] Li C., Jin Y., Wei S., Sun Y., Jiang L., Zhu Q., Farmer D.G., Busuttil R.W., Kupiec-Weglinski J.W., Ke B. (2019). Hippo Signaling Controls NLR Family Pyrin Domain Containing 3 Activation and Governs Immunoregulation of Mesenchymal Stem Cells in Mouse Liver Injury. Hepatology.

[151] Liu T., Wang L., Liang P., Wang X., Liu Y., Cai J., She Y., Wang D., Wang Z., Guo Z. (2021). USP19 suppresses inflammation and promotes M2-like macrophage polarization by manipulating NLRP3 function via autophagy. Cell. Mol. Immunol..

